# Enriching a biomedical event corpus with meta-knowledge annotation

**DOI:** 10.1186/1471-2105-12-393

**Published:** 2011-10-10

**Authors:** Paul Thompson, Raheel Nawaz, John McNaught, Sophia Ananiadou

**Affiliations:** 1 National Centre for Text Mining, Manchester Interdisciplinary Biocentre, School of Computer Science, University of Manchester, 131 Princess Street, Manchester, M1 7DN, UK

## Abstract

**Background:**

Biomedical papers contain rich information about entities, facts and events of biological relevance. To discover these automatically, we use text mining techniques, which rely on annotated corpora for training. In order to extract protein-protein interactions, genotype-phenotype/gene-disease associations, etc., we rely on event corpora that are annotated with classified, structured representations of important facts and findings contained within text. These provide an important resource for the training of domain-specific information extraction (IE) systems, to facilitate semantic-based searching of documents. Correct interpretation of these events is not possible without additional information, e.g., does an event describe a fact, a hypothesis, an experimental result or an analysis of results? How confident is the author about the validity of her analyses? These and other types of information, which we collectively term *meta-knowledge*, can be derived from the context of the event.

**Results:**

We have designed an annotation scheme for meta-knowledge enrichment of biomedical event corpora. The scheme is multi-dimensional, in that each event is annotated for 5 different aspects of meta-knowledge that can be derived from the textual context of the event. Textual clues used to determine the values are also annotated. The scheme is intended to be general enough to allow integration with different types of bio-event annotation, whilst being detailed enough to capture important subtleties in the nature of the meta-knowledge expressed in the text. We report here on both the main features of the annotation scheme, as well as its application to the GENIA event corpus (1000 abstracts with 36,858 events). High levels of inter-annotator agreement have been achieved, falling in the range of 0.84-0.93 Kappa.

**Conclusion:**

By augmenting event annotations with meta-knowledge, more sophisticated IE systems can be trained, which allow interpretative information to be specified as part of the search criteria. This can assist in a number of important tasks, e.g., finding new experimental knowledge to facilitate database curation, enabling textual inference to detect entailments and contradictions, etc. To our knowledge, our scheme is unique within the field with regards to the diversity of meta-knowledge aspects annotated for each event.

## Background

Due to the rapid advances in biomedical research, scientific literature is being published at an ever-increasing rate. This makes it highly important to provide researchers with automated, efficient and accurate means of locating the information they require, allowing them to keep abreast of developments within biomedicine [1-5]. Such automated means can be facilitated through text mining, which is receiving increasing interest within the biomedical field [6,7]. Text mining enriches text via the addition of semantic metadata, and thus permits tasks such as analysing molecular pathways [8] and semantic searching [9].

### Event-based text mining

Information extraction (IE) systems facilitate semantic searching by producing classified, structured, template-like representations of important facts and findings contained within documents, called *events*. As the features of texts and the types of events to be recognised vary between different subject domains, IE systems must be adapted to deal with specific domains. A well-established method of carrying out this adaptation is through training using annotated corpora (e.g., [10-12]). Accordingly, a number of corpora of biomedical texts annotated for events have been produced (e.g., [13-15]), on which IE systems in the biomedical domain can be trained.

Event annotation in these corpora typically includes the identification of the trigger, type and participants of the event. The *event trigger *is a word or phrase in the sentence which indicates the occurrence of the event, and around which the other parts of the event are organised. The *event type *(generally assigned from an ontology) categorises the type of information expressed by the event. The event participants, i.e., entities or other events that contribute towards the description of the event, are also part of the event representation, and are often categorised using semantic role labels such as CAUSE (i.e., the entity or other event that is responsible for the event occurring) and THEME (i.e., the entity or other event that is affected by the event) to indicate their contribution towards the event description. Events that contain further events as participants are often referred to as *complex *events, while *simple *events only contain entities as participants. Usually, semantic types (e.g. *gene*, *protein*, etc.) are also assigned to the named entities (NEs) participating in the event. Other types of participants are also possible, corresponding, for example, to the location or environmental conditions under which the event took place.

In order to illustrate this typical event representation, consider sentence (1).

*(1) The results suggest that the narL gene product activates the nitrate reductase operon*.

The typical structured representation of the biomedical event described in this sentence, is as follows:

EVENT-TRIGGER: *activates*

EVENT-TYPE: positive_regulation 

THEME: *nitrate reductase operon:*operon

CAUSE: *narL gene product*: protein

The automatic recognition of such structured events facilitates sophisticated semantic querying of documents, which provides much greater power than conventional search techniques. Rather than simply searching for keywords in documents, users can search for specific types of events in documents, through (partial) completion of a template. This template allows different types of restrictions to be placed on the events that are required to be found [9], e.g.,:

• The type of event to be retrieved.

• The types of participants that should be present in the event.

• The values of these participants, which could be specified in terms of either specific values or NE types.

The fact that event and NE types are often hierarchically structured can provide the user with a large amount of flexibility regarding the generality or specificity of their query.

### Event interpretation and the role of meta-knowledge

Despite the increased power and more focussed searching that event-based searching can provide over traditional keyword-based searches, typical event annotations do not capture contextual information from the sentence, which can be vital for the correct interpretation of the event [16]. Let us consider again sentence (1), and in particular the phrase at the beginning of the sentence, i.e., *The results suggest that *... This phrase allows us to determine the following about the event that follows:

• It is based on an analysis of experimental results.

• It is stated with a certain amount of speculation (according to the use of the verb *suggest*, rather than a more definite verb, such as *demonstrate*).

Altering the words in the context of the event can affect its interpretation in both subtle and significant ways. Consider the examples below:

*(2a) It is **known **that the narL gene product activates the nitrate reductase operon*.

*(2b) We **examined **whether the narL gene product activates the nitrate reductase operon*.

*(2c) The narL gene product did **not **activate the nitrate reductase operon*.

*(2d) These results **suggest **that the narL gene product **might **be activated by the nitrate reductase operon*.

(2e) The narL gene product **partially** activated the nitrate reductase operon

*(2f) **Previous studies **have shown that the narL gene product activates the nitrate reductase operon*.

If only the event type and participants are considered, then the events in sentences (2a-f) are identical to the event in sentence (1). However, the examples clearly illustrate that it is important to consider the context in which the event occurs, since a wide range of different types of information may be expressed that relate directly to the interpretation of the event.

In sentence (2a), the word *known *tells us that the event is a generally accepted fact, while in (2b), the interpretation is completely different. The word *examined *shows that the event is under investigation, and hence the truth value of the event is unknown. The presence of the word *not *in sentence (2c) shows that the event is negated, i.e. it did not happen. In sentence (2d), the presence of the word *might *(in addition to *suggest*) adds further speculation regarding the truth of the event. The word *partially *in (2e) does not challenge the truth of the event, but rather conveys the information that the strength or intensity of the event is less than may be expected by default. Finally, the phrase *previous studies *in (2f) shows that the event is based on information available in previously published papers, rather than relating to new information from the current study.

We use the term *meta-knowledge *to collectively refer to the different types of interpretative information available in the above sentences. There are several tasks in which biologists have to search and review the literature that could benefit from the automatic recognition of meta-knowledge about events. These tasks include building and updating models of biological processes, such as pathways [17] and curation of biological databases [18,19]. Central to both of these tasks is the identification of *new knowledge *that can enhance these resources, e.g., to build upon an existing, but incomplete model of a biological process [20], or to ensure that a database is kept up to date. New knowledge should correspond to experimental findings or conclusions that relate to the current study, which are stated with a high degree of confidence, rather than, e.g., more tentative hypotheses. In the case of an analytical conclusion, it may be important to find appropriate evidence that supports this claim [16] before allowing it to be added to the database.

Other users may be interested in checking for inconsistencies or contradictions in the literature. The identification of meta-knowledge could also help to flag such information. Consider, for example, the case where an event with the same ontological type and identical participants is stated as being true in one article and false in another. If the textual context of both events shows them to have been stated as facts, then this could constitute a serious contradiction. If, however, one of the events is marked as being a hypothesis, then the consequences are not so serious, since the hypothesis may have been later disproved. The automatic identification of meta-knowledge about events can clearly be an asset in such scenarios, and can prevent users from spending time manually examining the textual context of each and every event that has been extracted from a large document collection in order to determine the intended interpretation.

In response to the issues outlined above, we have developed a new annotation scheme that is specifically tailored to enriching biomedical event corpora with meta-knowledge, in order to facilitate the training of more useful systems in the context of various IE tasks performed on biomedical literature. As illustrated by the example sentences above, a number of different types of meta-knowledge may be encoded in the context of an event, e.g., general information type (fact, experimental result, analysis of results), level of confidence/certainty towards the event, polarity of the event (positive or negative), etc. In order to account for this, our annotation scheme is multi-dimensional, with each dimension encoding a different type of information. Each of the 5 dimensions has a fixed set of possible values. For each event, the annotation task consists of determining the most appropriate value for each dimension. Textual clue expressions that are used to determine the values are also annotated, when they are present.

Following an initial annotation experiment by two of the authors to evaluate the feasibility of the scheme [21], we applied our scheme to the complete GENIA event corpus [14]. This consists of 1000 MEDLINE abstracts, containing a total of 36,858 events. The annotation was carried out by two annotators, who were trained in the application of the scheme, and provided with a comprehensive set of annotation guidelines. The consistency and quality of the annotations produced were ensured though double annotation of a portion of the corpus.

To our knowledge, the enriched corpus represents a unique effort within the domain, in terms of the amount of meta-knowledge information annotated at such a fine-grained level of granularity (i.e., events). As the GENIA event corpus is currently the largest biomedical corpus annotated with events, the enrichment of this entire corpus with meta-knowledge annotation constitutes a valuable resource for training IE systems to recognise not only the core information about events and their participants, but also additional information to aid in their correct interpretation and provide enhanced search facilities. The corpus and annotation guidelines may be downloaded for academic purposes from http://www.nactem.ac.uk/meta-knowledge/.

### Related work

Although our approach to annotating multi-dimensional meta-knowledge information at the level of events is novel, the more general study of how knowledge in biomedical texts can be classified to aid in its interpretation is a well-established research topic. Two main threads of research can be identified, i.e.:

1) Construction of classified inventories of lexical markers (i.e., words or phrases) which can accompany statements to indicate their intended interpretation.

2) Production of corpora annotated with various different types of meta-knowledge at differing levels of granularity.

#### Lexical markers of meta-knowledge

The presence of specific cue words and phrases has been shown to be an important factor in classifying biomedical sentences automatically according to whether or not they express speculation [22,23]. Corpus-based studies of hedging (i.e., speculative statements) in biological texts [24,25] reinforce the above experimental findings, in that 85% of hedges were found to be conveyed lexically, i.e., through the use of particular words and phrases, rather than through more complex means, e.g., by using conditional clauses. The lexical means of hedging in biological texts have also been found to be quite different to academic writing in general, with modal auxiliaries (e.g., *may*, *could*, *would*, etc.) playing a more minor role, and other verbs, adjectives and adverbs playing a more significant role [24]. It has additionally been shown that, in addition to speculation, specific lexical markers can denote other types of information pertinent to meta-knowledge identification, e.g., markers of certainty [26], as well as deductions or sensory (i.e. visual) evidence [24].

Based on the above, we can determine that lexical markers play an important role in distinguishing several different types of meta-knowledge, and also that there is a potentially wide range of different markers that can be used. For example, [27] identified 190 hedging cues that are used in biomedical research articles. Our own previous work [28] on identifying and categorising lexical markers of meta-knowledge demonstrated that such markers are to some extent domain-dependent. In contrast to other studies, we took a multi-dimensional approach to the categorisation, acknowledging that different types of meta-knowledge may be expressed through different words in the same sentence. As an example, consider sentence (3).

*(3) The DNA-binding properties of mutations at positions 849 and 668 **may indicate **that the catalytic role of these side chains is associated with their interaction with the DNA substrate*.

Firstly, the word *indicate *denotes that the statement following *that *is to be interpreted as an analysis based on the evidence given at the beginning of the sentence (rather than, e.g., a well-known fact or a direct experimental observation). Secondly, the word *may *conveys the fact that the author only has a medium level of confidence regarding this analysis.

Although such examples serve to demonstrate that a multi-dimensional approach recognising meta-knowledge information is necessary to correctly capture potential nuances of interpretation, it is important to note that taking a purely lexical approach to recognising meta-knowledge is not sufficient (i.e., simply looking for words from lists of cues that co-occur in the same sentences as events of interest). The reasons for this include:

a) The presence of a particular marker does not guarantee that the "expected" interpretation can be assumed [29]. Some markers may have senses that vary according to their context. As noted in [30], "Every instance should ... be studied in its sentential co-text" (p.125).

b) Although lexical markers are an important part of meta-knowledge recognition, there are other ways in which meta-knowledge can be expressed. This has been demonstrated in a study involving the annotation of rhetorical zones in biology papers (e.g., background, method, result, implication, etc.) [31], based on a scheme originally proposed in [32]. An analysis of features used to determine different types of zone in the annotated papers revealed that, in addition to explicit lexical markers, features such as the main verb in the clause, tense, section, position in the sentence within the paragraph and presence of citations in the sentence can also be important.

Thus, rather than assigning meta-knowledge based only on categorised lists of clue words and expressions, there is a need to produce corpora annotated with meta-knowledge, on which enhanced IE systems can be trained. By annotating meta-knowledge information for each relevant instance (e.g., an event), regardless of the presence of particular lexical markers, systems can be trained to recognise other types of features that can help to assign meta-knowledge values. However, given that the importance of lexical markers in the recognition of meta-knowledge has been clearly illustrated, explicit annotation of such markers should be carried out as part of the annotation process, whenever they are present.

#### Existing corpora with meta-knowledge annotations

There are several existing corpora with some degree of meta-knowledge annotation. These corpora vary in both the richness of the annotation added, and the type/size of the units at which the meta-knowledge annotation has been performed. Taking the unit of annotation into account, we can distinguish between annotations that apply to continuous text spans, and annotations that have been performed at the event level.

Annotations applied to continuous text spans most often cover only a single aspect of meta-knowledge, and are most often carried out at the level of the sentence. The most common types of meta-knowledge annotated correspond to either speculation/certainty level, e.g., [22,23], or general information content/rhetorical intent, e.g., background, methods, results, insights, etc. This latter type of annotation has been attempted both on abstracts [33,34] and full papers [31,32,35], using schemes of varying complexity, ranging from 4 categories for abstracts, up to 14 categories for one of the full paper schemes. Accurate automatic categorisation of sentences in abstracts has been shown to be highly feasible [36], and this functionality has been integrated into the MEDIE intelligent search system [37].

A few annotation schemes consider more than one aspect of meta-knowledge. For example, the ART corpus and its CoreSC annotation scheme [38,39] augment general information content categories with additional attributes, such as *New *and *Old*, to denote current or previous work. The corpus described in [40] annotates both speculation and negation, together with their scopes. Uniquely amongst the corpora mentioned above, [40] also annotates the clue expressions (i.e. the negative and speculative keywords) on which the annotations are based.

Although sentences or larger zones of text [32] constitute straightforward and easily identifiable units of text on which to perform annotation, a problem is that a single sentence may express several different pieces of information, as illustrated by sentence (4).

(4) *Inhibition of the MAP kinase cascade with PD98059, a specific inhibitor of MAPK kinase 1, may prevent the rapid expression of the alpha2 integrin subunit*.

This sentence contains at least 3 distinct pieces of information:

• Description of an experimental method: *Inhibition of the MAP kinase cascade with PD98059*.

• A general fact: *PD98059 is a specific inhibitor of MAPK kinase 1*.

• A speculative analysis: *Inhibition of the MAP kinase may prevent the expression of the alpha2 integrin subunit*

The main verb in the sentence (i.e., *prevent*) describes the speculative analysis. In a sentence-based annotation scheme, this is likely to be the only information that is encoded. However, this means that other potentially important information in the sentence is disregarded. Some annotation schemes have attempted to overcome such problems by annotating meta-knowledge below the sentence level, i.e., clauses [41,42] or segments [43]. In the case of the latter scheme, a new segment is created whenever there is a change in the meta-knowledge being expressed. The scheme proposed for segments is more complex than the sentence-based schemes, in that it covers multiple types of meta-knowledge, i.e., focus (content type), polarity, certainty, type of evidence and direction/trend (either increase or decrease in quantity/quality). It has, however, been shown that training a system to automatically annotate along these different dimensions is highly feasible [44].

At the level of biomedical events, annotation of meta-knowledge is generally very basic, and is normally limited to negation, e.g., [15]. Negation is also the only attribute annotated in the corpus described in [45], even though a more complex scheme involving certainty, manner and direction was also initially proposed. To our knowledge, only the GENIA event corpus [14] goes beyond negation annotation, in that different levels of certainty (i.e. *probable *and *doubtful*) are also annotated.

Despite this current paucity of meta-knowledge annotation for events, our earlier examples have demonstrated that further information can usefully be specified at this level, including at least the general information content of the event, e.g. fact, experimental observation, analysis, etc. A possibility would be to "inherit" this information from a system trained to assign such information at the text span level (e.g. sentences or fragments), although this would not provide an optimal solution. The problem lies in the fact that text spans constitute continuous stretches of text, but events do not. The different constituents of an event annotation (i.e., trigger and participants) can be drawn from multiple, discontinuous parts of a sentence. There are almost always multiple events within a sentence, and the different participants of a particular event may be drawn from multiple sentence fragments. This means that mapping between text span meta-knowledge and event-level meta-knowledge cannot be carried out in a straightforward manner. Thus, for the purposes of training more sophisticated event-based information search systems, annotation of meta-knowledge directly at the event level can provide more precise and accurate information that relates directly to the event.

Based on the above findings, we embarked upon the design of an event-based meta-knowledge annotation scheme specifically tailored for biomedical events. In the remainder of this paper, we firstly cover the key aspects of this annotation scheme, followed by a description of the recruitment and training of annotators. We follow this by providing detailed statistics, results and evaluation of the application of the scheme to the GENIA event corpus. Finally, we present some conclusions and directions for further research.

## Methods

In this section, we begin by providing a general overview of our annotation scheme, followed by a more detailed description of each annotation dimension. Following a brief overview of the software used to perform the annotation, we describe how we conducted an annotation experiment to test the feasibility and soundness of our scheme, prior to beginning full-scale annotation. The section concludes with a brief explanation of the recruitment and training of our annotators.

### Meta-knowledge annotation scheme for events

The aim of our meta-knowledge scheme is to capture as much useful information as possible that is specified about individual events in their textual context, in order to support users of event-based search systems in a number of tasks, including the discovery of new knowledge and the detection of contradictions. In order to achieve this aim, our annotation scheme identifies 5 different dimensions of information for each event, taking inspiration from previous multi-dimensional schemes (e.g. [39,43,45]). In addition to allowing several distinct types of information to be encoded about events, a multi-dimensional scheme is advantageous, in that the interplay between the different dimension values can be used to derive further useful information (*hyper-dimensions) *regarding the interpretation of the event.

Each dimension of the meta-knowledge scheme consists of a set of complete and mutually-exclusive categories, i.e., any given bio-event belongs to exactly one category in each dimension. The set of possible values for each dimension was determined through a detailed study of over 100 event-annotated biomedical abstracts. In order to minimise the annotation burden, the number of possible categories within each dimension has been kept as small as possible, whilst still respecting important distinctions in meta-knowledge that have been observed during our corpus study. Due to the demonstrated importance of lexical clues in the identification of certain meta-knowledge categories, the annotation task involves identifying such clues, when they are present.

**Figure 1:**
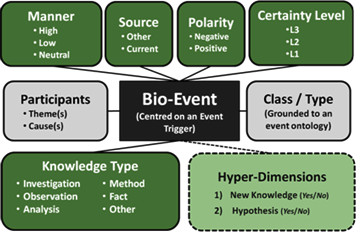
**Meta-knowledge annotation scheme**. The boxes with the grey background correspond to information that is common to most bio-event annotation schemes, i.e., the participants in the event, together with an indication of the class or type of the event. The boxes with the dark green backgrounds correspond to our proposed meta-knowledge annotation dimensions and their possible values, whilst the light green box shows the hyper-dimensions that can be derived by considering a combination of the annotated dimensions.

Figure 1 provides an overview of the annotation scheme. Below, we provide a brief description of each annotation dimension. Further details and examples are provided in the comprehensive (66-page) annotation guidelines, which are available at: http://www.nactem.ac.uk/meta-knowledge/Annotation_Guidelines.pdf

#### Knowledge Type (KT)

This dimension is responsible for capturing the general information content of the event. The type of information encoded is at a slightly different level to some of the comparable sentence-based schemes, which have categories relating to structure or "zones" within a document, e.g. *background *or *conclusion*. Rather, our KT dimension attempts to identify a small number of more general information types that can be used to characterise events, regardless of the zone in which they occur. As such, our scheme can be seen as complementary to structure or zone-based schemes, providing a finer-grained analysis of the different types of information that can occur within a particular zone. The KT features we have defined are as follows:

• **Investigation: **Enquiries or investigations, which have either already been conducted or are planned for the future, typically accompanied by lexical clues like *examined*, *investigated *and *studied*, etc.

• **Observation: **Direct observations, sometimes represented by lexical clues like *found*, *observed *and *report*, etc. Event triggers in the past tense typically also describe observations.

• **Analysis: **Inferences, interpretations, speculations or other types of cognitive analysis, always accompanied by lexical clues, typical examples of which include *suggest*, *indicate*, *therefore *and *conclude*, etc.

• **Method: **Events that describe experimental methods. Denoted by trigger words that describe experimental methods, e.g., *stimulate*, *addition*.

• **Fact: **Events that describe general facts and well-established knowledge, typically denoted by present tense event triggers that describe biological processes, and sometimes accompanied by the lexical clue *known*.

• **Other: **The default category, assigned to events that either do not fit into one of the above categories, do not express complete information, or whose KT is unclear or is assignable from the context. These are mostly *non-propositional *events, i.e., events which cannot be ascribed a truth value due to lack of available (contextual) information.

#### Certainty Level (CL)

This dimension aims to identify the level of certainty associated with occurrence of the event, as ascribed by the authors. It comes into play whenever there is explicit indication that there is less than complete confidence that the specified event will occur. This could be because:

• There is uncertainty regarding the general truth value ascribed to the event.

• It is perceived that the event may not take place all of the time.

Different degrees of uncertainty and frequency can be considered as points on a continuous scale, and there is an ongoing discussion regarding whether it is possible to partition the epistemic scale into discrete categories [42]. However, the use of a number of distinct categories is undoubtedly easier for annotation purposes and has been proposed in a number of previous schemes. Although recent work has suggested the use of four or more categories [28,42,44], our initial analysis of bio-event corpora showed that only three levels of certainty seem readily distinguishable for bio-events. This is in line with [46], whose analysis of general English showed that there are at least three articulated points on the epistemic scale.

Like the scheme described in [43], we have chosen to use numerical values for the CL dimension, in order to reduce potential annotator confusions or biases that may be introduced through the use of labels corresponding to particular lexical markers of each category, such as *probable *or *possible*. Such labels could in any case be misleading, given that frequency can also come into play in assigning the correct category. Our chosen values of the CL dimension are defined as follows:

• **L3: **The default category. **No **explicit expression that either:

(a) There is uncertainty or speculation towards the event.

(b) The event does not occur all of the time.

• **L2: **Explicit indication of either:

*(a) *High (but not complete) confidence or slight speculation towards the event. Typical lexical clues include *likely*, *probably*, *suggest *and *indicate*.

(b) The event occurs frequently, but not all of the time. Typical lexical clues include *normally*, *often*, *frequently*.

• **L1**: Explicit indication of either:

*(a) *Low confidence or considerable speculation towards the event. Typical lexical clues include *may*, *might *and *perhaps*.

(b) The event occurs infrequently or only some of the time. Typical lexical markers may include *sometimes*, *rarely*, *scarcely*, etc.

#### Polarity

This dimension has been designed to capture the truth value of the assertion encapsulated by the event. We define a negated event as one that describes the absence or non-existence of an entity or a process. That is to say, the event may describe that a process does not or did not happen, or that an entity is absent or does not exist. The recognition of such information is vital, as the interpretation of a negated event instance is completely opposite to the interpretation of a non-negated (positive) instance of the same event. Our scheme permits the following two values for this dimension:

• **Positive: **No explicit negation of the event (default)

• **Negative: **The event has been negated according to the description above. The negation may be indicated through lexical clues such as *no*, *not*, *fail*, *lack*, etc.

#### Manner

This dimension identifies the rate, level, strength or intensity of the event (in biological terms). Such information has previously been shown to be relevant for biologists. The event annotation scheme for the GREC corpus [13], which was designed in consultation with biologists, identified expressions of manner as one of the semantic roles associated with event. The proposal for the annotation of protein-protein interactions suggested in [45] also lists manner as a potentially useful attribute to annotate. Inspired by these works, we build upon the types of manner annotation available in the GREC corpus by adopting a three-way categorisation of manner, as shown below:

• **High: **Explicit indication that the event occurs at a high rate, level, strength or intensity. Clue expressions are typically adjectives or adverbs such as *high*, *strongly*, *rapidly*, *potent*, etc.

• **Low: **Explicit indication that the event occurs at a low rate, level, strength or intensity. Clue expressions are typically adjectives and adverbs such as *slightly*, *partially*, *small*, etc.

• **Neutral: **The default category. Assigned when there is no explicit indication of either high or low manner, but also in the rare cases when neutral manner is explicitly indicated, using clue words such as *normal *or *medium*, etc.

#### Source

This dimension denotes the source or origin of the knowledge being expressed by the event. Specifically, we distinguish between events that can be attributed to the current study, and those that are attributed to other studies. Information about knowledge source has been demonstrated to be important through its annotation in both the Gene Ontology [18] and in the corpora presented in [38] and [43]. This dimension can help in distinguishing new experimental knowledge from previously reported knowledge. Two possible values are distinguished, as follows:

• **Other: **The event is attributed to a previous study. In this case, explicit clues are normally present, and can be indicated either by the use of clue words such as *previously*, *recent studies*, etc., or by the presence of citations.

• **Current: **The event makes an assertion that can be attributed to the current study. This is the default category, and is assigned in the absence of explicit lexical or contextual clues, although explicit clues such as *the present study *may be encountered.

#### Hyper-Dimensions

A defining feature of our annotation scheme is the fact that, in addition to the explicitly annotated dimensions, further information can be inferred by considering combinations of some of these dimensions. We refer to these additional types of information as the *hyper-dimensions *of our scheme, of which we have identified two.

• **New Knowledge - **The isolation of events describing new knowledge is, as we have described earlier, important for certain tasks undertaken by biologists. However, it is not possible to determine whether an event represents new knowledge by considering only a single annotation dimension. For example, events that have been assigned *KT = Observation *could correspond to new knowledge, but only if they represent observations from the current study, rather than observations cited from elsewhere. In a similar way, a *KT = Analysis *event drawn from experimental results in the current study could be treated as new knowledge, but generally only if it represents a straightforward interpretation of results, rather than something more speculative. Thus, we consider *New Knowledge *to be a hyper-dimension, whose value (either *Yes *or *No*) can be inferred by considering a combination of the values assigned to the *KT*, *Source *and *CL *dimensions. Table 1 is an inference table that can be used to obtain the appropriate value for *New Knowledge*, based on the values assigned to the three dimensions mentioned above.

**Table 1:** Inference table for *New Knowledge *hyper-dimension

Source (Annotated)	KT (Annotated)	CL (Annotated)	New Knowledge (Inferred)
Other	X	X	No
X	X	L2	No
X	X	L1	No
Current	Observation	L3	Yes
Current	Analysis	L3	Yes
X	Fact	X	No
X	Method	X	No
X	Other	X	No
X	Investigation	X	No

The symbol 'X' indicates a "don't care condition", meaning that this value does not have any impact on the result

• **Hypothesis - **The binary value of this hyper-dimension can be inferred by considering the values of the *KT *and *CL *dimensions. Events with a *KT *value of *Investigation *can always be assumed to be a hypothesis. However, if the KT value is *Analysis*, then only those events with a *CL *value of L1 or L2 (speculative inferences made on the basis of results) should be considered as hypotheses, to be matched with more definite experimental evidence when available. A value of L3 in this instance would normally be classed as an instance of new knowledge, as indicated in Table 1. The cases in which an event can be assumed to be a hypothesis are summarised in Table 2.

**Table 2:** Inference table for *Hypothesis *hyper-dimension

KT (Annotated)	CL (Annotated)	Hypothesis (Inferred)
Fact	X	No
Method	X	No
Other	X	No
Observation	X	No
Analysis	L3	No
Analysis	L2	Yes
Analysis	L1	Yes
Investigation	X	Yes

The symbol 'X' indicates a "don't care condition", meaning that this value does not have any impact on the result.

### Annotation software

The original annotation of the GENIA event corpus was performed using the X-Conc suite [47]. This is a collection of XML-based tools that are integrated to support the development and annotation of corpora, running as a Java plug-in within the Eclipse software development platform [48]. Customising the information to be annotated and the way in which it is displayed is controlled completely through XML DTD and stylesheet (CSS) files. We decided to use this tool to carry out meta-knowledge annotation of events in the GENIA event corpus, as only minimal customisation of the existing DTD and CSS files would be required.

### Testing of annotation scheme

Prior to annotation of the full GENIA event corpus, a small annotation experiment was conducted to verify the feasibility and soundness of the meta-knowledge annotation scheme [21]. Two of the authors independently applied the annotation scheme to 70 abstracts selected at random from the GENIA pathway corpus [49], using the annotation manual we had developed. The experiment helped to demonstrate the soundness of both the scheme itself and the guidelines, given that Kappa scores [50] scores of 0.89 - 0.95 were achieved. Also, the fact that all categories within all dimensions were annotated, at least to a certain extent, suggested that none of the proposed categories was redundant.

### Annotators and training

In order to ensure the efficacy of the guidelines and the reproducibility of the annotation task, we recruited 2 external annotators to carry out the annotation of our gold standard corpus. An important consideration was the type of expertise required by the annotators. It has previously been found that at least negations and speculations in biomedical texts can be reliably detected by linguists [40]. The scope of our meta-knowledge annotation is wider, involving some scientifically motivated aspects (i.e., *KT *and *Manner*), but the assignment of certain dimension values is somewhat linguistically oriented, e.g., it is often the case that clue expressions have a grammatical relationship to the event trigger that they modify. In order to verify the extent to which either domain-specific biological knowledge or linguistic knowledge is required to perform the annotation accurately, we recruited a biology expert and a linguistics expert to carry out the task. Both annotators have near-native competency of English, which we considered to be important to carry out the task accurately.

The annotators undertook training prior to commencing the annotation of the gold standard corpus. This training began with initial introductory sessions, in which the annotation scheme and guidelines were explained, and the X-Conc annotation tool was demonstrated. Subsequently, the annotators carried out practice annotation tasks. For this purpose, we used the same corpus of 70 abstracts from the GENIA pathway corpus that was used to test the feasibility of the scheme, as described above. Both annotators were given the same sets of abstracts to annotate, independently of each other. This allowed us to detect a maximal number of potential annotation errors and discrepancies produced by the annotators, as we could conduct comparisons not only between the annotators themselves, but also against a gold standard corpus. The annotators returned a set of abstracts each week, in response to which we produced detailed feedback reports highlighting annotation errors. These reports were thoroughly discussed with the annotators, in order to maximally enhance and accelerate the learning process. Often, errors made by the annotators revealed potential problems with the annotation guidelines, which were addressed by updating the guidelines accordingly.

## Results and Discussion

In this section, we firstly provide key statistics regarding the meta-knowledge annotations produced, together with a brief discussion regarding the salient characteristics of the corpus. This is followed by a report on the level of agreement achieved between the annotators in the double-annotated part of the corpus, and an examination of the different kinds of discrepancies that were found within these abstracts.

### General corpus characteristics

Below, we discuss the general distribution of the annotations amongst the different categories for each dimension, and also provide lists of the most commonly annotated clue expressions.

#### Knowledge Type (KT)

Table 3 shows the number of instances of each category annotated for the *Knowledge Type *dimension. The most common category is *Observation*, constituting just over a third of the total number of events. This result is unsurprising, since abstracts would be expected to focus mainly on definite experimental observations and results, both of which fall into this category. The *Other *category is almost as common as *Observation*. Such events are generally the participant events of *Investigation*, *Analysis *or *Fact *events which, out of the context of their parent event, have no specific KT interpretation. The total number of *Other *events is very similar to the combined total of *Investigation*, *Analysis *and *Method *events. This is to be expected, given the high proportion (44%) of complex events present in the corpus.

**Table 3:** Distribution of annotated categories for *Knowledge Type (KT)*

Category	Freq	% of total events
Observation	12821	34.7%
Other	11537	31.3%
Analysis	6578	17.8%
Fact	2998	8.1%
Investigation	1948	5.3%
Method	976	2.6%

The proportion of *Analysis *events is much smaller but still quite significant, since most abstracts contain at least some analysis of the experimental results obtained. The usual inclusion of a small amount of background factual information to put the current study into context accounts for the average of 3 events per abstract (8% of all events) that are assigned the *Fact *category. Even briefer are the descriptions of what is to be investigated, with an average of 2 *Investigation *events per abstract (5% of all events). The scarcity of events describing methods (2.6% of events, or less than 1 event per abstract) shows that providing details of experimental setup is very rare within abstracts.

Table 4 shows the most commonly annotated clue expressions for the *KT *categories of *Analysis*, *Investigation *and *Observation*. Clues were also annotated for the *Fact *category, if they were present. However, only 139 of the 2998 *Fact *events (4.6%) have a clue expression annotated. Of these annotated clue expressions, 106 (76%) correspond to the word *known*. Clue expression annotation was also optional for the *Observation *category, in which only 937 (7.3%) of the total number of events are accompanied by a clue. For the *Investigation *and *Analysis *categories, all annotated events have a clue expression.

**Table 4:** Most common *KT *clue expressions

**Analysis Clue**	**Freq**	**Investigation Clue**	**Freq**	**Observation Clue**	**Freq**
		
suggest	408	examined	207	found	361
show	353	investigated	205	observed	226
demonstrate	335	analyzed	119	detected	141
demonstrated	332	studied	94	detectable	48
showed	246	to determine	50	seen	32
shown	244	tested	39	noted	17
may	242	measured	25	find	11
can	232	monitored	25	detect	11
associated	215	to investigate	23	findings	11
indicate	211	to examine	21	observations	9
revealed	196	to study	21	finding	9
suggesting	140	analysis	20	show	6
report	114	studies	20	report	6
identified	112	to identify	16	exhibit	5
thus	108	investigate	15		

For both *Investigation *and *Observation*, the top three most common clue expressions are past tense verbs, while the use of the present tense appears to be more dominant for describing *Analysis *events. The use of infinitive forms (e.g., *to investigate*) as clues seems to be a particular feature of the *Investigation *category. Whilst most clues are verbal forms, words with other parts of speech can sometimes constitute reliable clues (e.g., *thus *for *Analysis*, and *detectable *for *Observation*).

#### Certainty Level (CL)

The distribution of *CL *annotations is shown in Table 5. Despite the relative scarcity of *CL *marking on events, it should be noted that this dimension is only applicable when the *KT *= *Analysis*. Taking this into consideration, the need for this dimension becomes more apparent: whilst over half of *Analysis *events (54.7%) are stated with no uncertainty, this also means that almost half of these events *do *express some kind of uncertainty. In fact, approximately one third (33.7%) of all *Analysis *events are annotated as *CL *= *L2*, whilst 11.6% are reported with less certainty (*CL *= *L1*). The very nature of abstracts means that the high proportion of events with no uncertainty is to be expected. As authors aim to "sell" the most positive aspects of their work in abstracts, it makes sense that the majority of analyses should be presented in a confident manner.

**Table 5:** Distribution of annotated categories for *Certainty Level (CL)*

Category	Freq	% of total events
L3 (default)	33876	91.9%
L2	2216	6.0%
L1	766	2.1%

However, the marking of slight uncertainty is sometimes necessary. The author's analyses of experimental results may have produced important outcomes, but yet they are not confident that their analysis is completely reliable. As stated in [24], "Scientists gain credibility by stating the strongest claims they can for their evidence, but they also need to insure against overstatement." (p. 257). Such insurance can often be achieved by the use of slight hedging (*CL = L2*). Greater speculation (*CL = L1*) is less common, as such credibility is reduced in this case.

As part of the original GENIA event annotation, *Uncertainty *was annotated as an event attribute. The default value is *Certain *and the other two values are *Probable *and *Doubtful*. In the GENIA event annotation guidelines, these attributes do not have clear definitions. However, *Probable *can be defined loosely as something that is hypothesized by the author, while *Doubtful *is something that is investigated. *Probable *has more in common with our CL dimension, while *Doubtful *is more closely linked to the *Investigation *category of our KT dimension. Therefore, the GENIA *Uncertainty *attribute does not distinguish between degrees of uncertainty in the same way as our meta-knowledge scheme. Comparison of results confirms this - of the events annotated with *Uncertainty = Probable*, there are similar proportions of events that have been annotated with *CL = L1 *(33.6% of *Probable *events) and *CL = L2 *(42.2% of *Probable *events). It is also worth noting that the total percentage of events identified with some degree of uncertainty using our scheme (*CL = L1 *or *CL = L2*) is 8.1%. This is almost double the percentage of events annotated as *Probable *(4.3% of all events), showing that our more detailed guidelines for CL annotation have helped to identify a far greater number of events expressing some degree of speculation.

Discrepancies can also be found regarding the *Doubtful *category. Events annotated with this category constitute 3.7% of all events in the corpus. Whilst, as expected, the vast majority of these correspond to events that have been annotated as *KT = Investigation *in our meta-knowledge scheme (1022 out of a total of 1349 *Doubtful *events, i.e. 75.8%), some *Doubtful *events also correspond to events with other *KT *values (most notably *Analysis *with *CL *values of *L3*, *L2 *or *L1*, which can also occur within the *Probable *category). This provides evidence that the boundary between *Doubtful *and *Probable *may not always have been clear to GENIA corpus annotators. In addition, our scheme identified 1948 events (5.3% of all events) with *KT = Investigation*, meaning that there were some 900 investigative events, i.e., 2.4% of all events, which were not identified during the original GENIA event annotation.

Table 6 shows the most commonly annotated clue expressions for the L2 and L1 values. For L2, the most common expression is *can*, which normally expresses ability rather than speculation (together with the clues *ability *and *able*). If an event has the ability to occur, then there is no guarantee that it will occur all of the time, and hence it is sensible that the event should be annotated as having less than complete certainty.

**Table 6:** Most common CL clue expressions

**L2 Clue**	**Freq**	**L3 Clue**	**Freq**
	
can	407	may	516
suggest	285	might	75
indicate	150	could	55
suggesting	112	possible	32
ability	108	potential	23
indicated	99	possibility	10
appears	88	possibly	10
able	86	potentially	10
indicating	72	perhaps	5
likely	52	propose	4

All of the other words in the L2 list express slight speculation or hedging, mostly corresponding to different forms of the verbs *suggest *and *indicate*. In Table 4, it was seen that these verbs also rank amongst the most common *Analysis *clues, showing that it is common for analysis and slight speculation to be simultaneously expressed using a single clue word. For the indication of L1 certainty, modal auxiliary verbs are particularly common, with *may *accounting for 67.4% of all annotated L1 clues, and *might *and *could *constituting a significant proportion of the remainder. The L1 category has a very small number of distinct clue expressions (23), compared to 121 distinct expressions for L2.

#### Polarity

As seen in Table 7, only a small number of events are negated (6.1%). However, it is vital that such information is detected, as negation completely alters the meaning of the event. In the GENIA event corpus, negation is an aspect of meta-knowledge that was annotated as part of the original annotation (via the *assertion *attribute). There is almost, but not complete agreement, between *Polarity = Negative *and *assertion = non-exist*, with a total of 2262 events (6.1% of all events) annotated with the former and 2351 (6.4% of all events) in the latter case. The slightly fewer negative annotations produced by our annotation are mainly due to the fact that some events annotated as negative in the original GENIA annotation actually convey low manner. An example is shown in sentence (5). In this and following examples, the event trigger is shown in small capitals and the clue expression is emboldened.

**Table 7:** Distribution of annotated categories for *Polarity*

Polarity	Freq	% of total events
Positive (default)	34595	93.9%
Negative	2263	6.1%

(5) *AP-1 but not NF-IL-6 DNA binding activity was also detected in C5a-stimulated PBMC; however, its delayed expression (maximal at 4 hours) suggested a **less important*** ROLE* in the rapid production of IL-8*.

The event encodes the fact that the expression of AP-1 only has a minor role in the rapid production of IL-8. As the GENIA annotation had no special means to encode that an event has low intensity or impact, the original annotator chose to annotate it as a negative event, even though this is not strictly correct. Our annotation scheme, with its *Manner *dimension, allows the subtle difference between an event having a low impact and an event not happening at all to be encoded. Our scheme annotates low impact events such as the above with *Polarity = Positive *but *Manner = Low*.

In Table 8, we examine the distribution of negated events amongst the different KT categories. Although negated events occur within events belonging to all KT categories, the distribution is quite uneven. Only observations and analyses are negated with any amount of regularity. Events belonging to the remaining KT values are virtually always expressed with positive polarity, with only around 3.5% of fact-bearing events being negative, and the other three categories (*Investigation*, *Method *and *Other*) only averaging one negative instance per hundred events.

**Table 8:** Distribution of negated events among *KT *categories

KT Category	Negated events (% within category)
Observation	1364 (10.6%)
Analysis	577 (8.7%)
Fact	105 (3.5%)
Other	187 (1.6%)
Method	10 (1.0%)
Investigation	20 (1.0%)

The low occurrence of negative instances amongst events with *KT = Investigation *events is quite intuitive - it is the norm to investigate why/whether something *does *take place, although in some instances there can be investigation into why something does not take place, such as in response to a previous negative finding, such as in (6).

(6) ***To determine ****why alveolar macrophages **do not*** EXPRESS* AP-1 DNA binding activity*, ...

Also, for methods, it is highly unusual to say that a particular method was not applied, unless in contrast to the case where the method *was *applied, as is the case in (7).

(7) *For comparison, we recruited a control group consisting of 32 healthy males and females with similar age distribution and **without** a history of* EXPOSURE *to MTBE or benzene*.

Table 9 displays the most commonly annotated clue expressions for negated events. Although the number of events we have identified as negated is roughly similar to those originally annotated in the GENIA event corpus, our annotation has the advantage of having identified a suitable clue expression for each negated event.

**Table 9:** Most common clue expressions for *Polarity = Negative*

Category	Freq
not	1141
no	199
independent	113
without	65
failed	47
nor	47
absence	42
neither	38
unaffected	28
lack	23
un	23
unable	19
independently	18
resistant	15
fails	13

The word *not *constitutes around half of all clue expressions for negation (50.4%), and is over 5 times more common than the next most common clue expression, *no*. Although most of the words in the list have an inherently negative meaning, the third most common word, i.e., *independent *(together with its associated adverb *independently*), does not. Closer examination shows that this negative meaning is quite context-dependent, in that it only denotes a negative meaning for events of type *Correlation *and *Regulation *(together with its sub-type *Positive_Regulation)*. For *Regulation *events, a typical example is shown in (8).

(8) *An alteration in the E2F-4 profile was ***INDEPENDENT*** of viral gene expression*.

In (8), the word *independent *acts as both the event trigger and the negative clue expression. The event denotes the fact that the alteration in the E2F-4 profile was not dependent on viral gene expression occurring. In other words, it is not the case that viral gene expression regulates the alteration in the E2F-4 profile. Events of type *Correlation *are annotated when there is some kind of association that holds between entities and/or other events. Sentence (9) shows an example of both a positive *Correlation *event and a negated *Correlation *event.

(9) *LPS-*INDUCED* NF-kappaB activation is protein tyrosine kinase *DEPENDENT* and protein kinase C ***INDEPENDENT**

There are three relevant events in (9). Firstly, the word *induced *is the trigger for a *Positive_Regulation *event in which *NF-kappaB activation *is regulated by *LPS*. The word *dependent *is the trigger for the second event, which is a positive *Correlation *event. It shows that that there is and association between the *Positive_Regulation *event and the protein *tyrosine kinase*. In contrast, the third event, triggered by *independent*, shows that no such association holds between the *Positive_Regulation *event and the protein *kinase C*. Hence, this is a negated *Correlation *event.

Some less commonly occurring negative clue expressions also only have negative meanings in very specific contexts. Consider (10).

(10) *These cells are *DEFICIENT *in FasL expression and apoptosis induced upon TCR triggering, although their cytokine (IL-2 and IFN-gamma) production is*** NORMAL**.

In (10), the word *deficient *indicates a *Negative_Regulation *event. However, the word *normal *indicates that no such negative regulation occurs in the case of IL-2 and IFN-gamma production. In the few contexts that *normal *occurs as a negative polarity marker, it is used in similar contexts, i.e., to contrast with a previously stated *Negative_Regulation *event. The word *silent *appears to be usable in similar contexts to negate events of type *Positive_Regulation*, in contrast to a positive occurrence of such an event.

#### Manner

As shown in Table 10, almost 5% of all events express a *Manner *value other than *Neutral*, which makes it only a slightly less commonly expressed phenomenon than negation. In the previous section, it has already been illustrated that the *Low *manner value can help distinguish between truly negative events, and those that occur at a low level or with low intensity. However, instances of *High *manner are much more common, and account for 81% of events for which there is an explicit indication of Manner.

**Table 10:** Distribution of annotated categories for *Manner*

Manner	Freq	% of total events
Neutral (default)	35143	95.3%
High	1392	3.8%
Low	323	0.8%

The distribution of events annotated with either *High *or *Low *Manner according to the KT value of the event is shown in Table 11. For the *Observation *category, explicit expression of *Manner *is observed in close to 1 in 10 events, making its frequency similar to the expressions of negation within this category. Of all events annotated for *Manner*, 66.5% correspond to those with the KT type of *Observation*. This makes it clear that a major usage of *Manner *marking is to refine the descriptions of experimental observations and results.

**Table 11:** Distribution of events with explicit *Manner *annotated among *KT *categories

KT Category	Events with *High* or* Low *Manner annotated (% within category)
Observation	1141 (8.9%)
Analysis	276 (4.2%)
Fact	120 (4.0%)
Other	171 (1.5%)
Investigation	5 (0.2%)
Method	2 (0.2%)

Table 12 shows the most commonly annotated clue expressions for both the *High *and *Low *values of the *Manner *dimension. In both cases, most of the clue expressions consist of adjectives or adverbs, with a range of meanings referring to degree (e.g., *completely*), speed or rate (e.g., *rapidly*), strength or intensity (e.g., *strongly*) and level (e.g. *high*). These differences in meaning of the manner expressions can be explained by the varying semantics of the biological processes that are described by events. In most cases, items in the *High *manner list have counterparts in the *Low *list, e.g., *significant *vs. *little*, *high *vs. *low*, *strongly *vs. *weakly*, *completely *vs. *partially*. It is notable that a counterpart of *rapidly *(e.g., *slowly*) appears to be missing from the list of *Low *clue expressions.

**Table 12:** Most common *Manner *clue expressions

**High Manner Clue**	**Freq**	**Low Manner Clue**	**Freq**
	
significantly	140	little	22
potent	84	low	15
markedly	81	little or no	13
rapidly	73	low levels	11
strongly	72	weak	11
rapid	65	limited	10
significant	39	low level	9
completely	36	weakly	9
strong	30	minimal	8
high	28	only a partial	8
high levels	28	no significant	8
overexpression	26	partially	8
highly	23	barely	7
marked	23	to a lesser extent	6
dramatically	22	not significant	6

In the *High *manner clue word list, a notable item is *overexpression*. Unlike the other clues in the list, which are independent of event type, this word is specific to events of type *Gene_Expression*, as it combines the meaning of the event type with the expression of *High *manner. Comparable examples appear very rare.

Some of the annotated clues for both *High *and *Low *manner contain numerical values, meaning that a pattern matching approach may be required when trying to recognise them in unseen texts. For example, the expression *n-fold *is often used to denote *High *Manner (often preceding the word *increase *or *decrease*), where *n *may be any numeric value. In other cases, *by n% *may follow one of these words. To indicate *Low *manner, the expressions *n-fold less *or *n-fold lower *are sometimes used.

#### Source

Regarding the *Source *dimension, only 1.5% of events in total have any evidence that they come from a source other than the current study, as shown in Table 13. This low percentage may be expected, given that abstracts are meant to summarise the work carried out in the current study. In addition, citations, which are a common way to denote previous work, are often not allowed within abstracts. It should be noted that a considerably greater proportion of events marked as *Source = Other *would be expected when applying the scheme to full papers, in which the *Background *section will normally contain a large number of references to and descriptions of previous work. Of the events annotated as *Source = Other *with abstracts, the vast majority (86%) have a KT value of *Analysis*.

**Table 13:** Distribution of annotated categories for *Source*

Source	Freq	% of total events
Current (default)	36313	98.5%
Other	545	1.5%

Table 14 shows the most commonly annotated clue expressions for *Source = Other*. Most of these consist of the words *previous *or *recent*, or phrases containing these words. The use of the passive voice with the present perfect tense (e.g. *has been studied) *is another common means to indicate that an event has previously been completed (e.g., in a previous study), but yet has relevance to the current study. This explains the relatively high occurrence of *has been *and *have been *as clues for *Source = Other*.

**Table 14:** Most common clue expressions for *Source = Other*

Clue	Freq
previously	118
has been	89
recently	67
have been	39
previous studies	24
recent studies	17
recent	15
previous	14
our previous studies	10
earlier	6

#### Hyper-dimensions

Using the inference tables discussed earlier (i.e., Table 1 and Table 2), we calculated the frequencies for the two hyper-dimensions, which are shown in Table 15. As part of the annotation carried out in [51], sentences containing descriptions of claims of new knowledge were annotated in both chemistry and computational linguistics research articles. The results showed that the proportion of sentences containing new knowledge was 63% for the chemistry articles and 72% for the computational linguistics articles. It may be expected that the amount of new knowledge presented in biomedical research articles would be more similar to chemistry articles than computational linguistics ones. However, the proportion of *events *that represent new knowledge in our corpus is somewhat lower than the proportion of sentences that contain new knowledge in chemistry. This lower percentage can be explained in a number of ways. Firstly, unlike our scheme, [51] treat experimental methods as new knowledge, and these make up a significant proportion of the new knowledge in the chemistry articles. In any case, as has been reported above, abstracts have a different structure to articles, and experimental methods are rarely reported. In addition, our *New Knowledge *hyper-dimension takes certainty level into account, and excludes events that are highly speculative. However, certainty level is not taken into account in [51]. Finally, the granularity of the schemes is different. Whilst [51] annotates at sentence level, our annotation is at the event level, of which there are an average of 3 to 4 per sentence. As some of these events represent non-propositional information, which cannot be treated as new knowledge, it makes sense that the proportion of events that represent new knowledge would be lower than the percentage of sentences that contain such information.

**Table 15:** Distribution of categories for the two hyper-dimensions

Hyper-dimension	Category	Freq	% of total events
New Knowledge	Yes	15985	43.4%
	No	20873	56.6%

Hypothesis	Yes	4924	13.4%
	No	31934	86.6%

### Inter-annotator agreement

In order to ensure the consistency and quality of the meta-knowledge annotation throughout the corpus, 104 randomly selected abstracts (10% of the entire corpus) were annotated by both annotators. This allowed us to calculate their agreement rates, in terms of Kappa values. The results for each dimension are reported in Table 16.

**Table 16:** Inter-annotator agreement rates

Dimension	Kappa value
Polarity	0.929
Source	0.878
CL	0.864
Manner	0.864
KT	0.843

High levels of agreement were achieved in each annotation dimension, with generally only very small differences between the agreement rates for different dimensions. This provides strong evidence that consistent annotation of meta-knowledge is a task that can be reliably undertaken by following the annotation guidelines, regardless of background (biology or linguistics).

The *Polarity *dimension has the highest rates of agreement. This could be because it is one of the two dimensions that have only two possible values (together with *Source*, which has the second highest agreement rate). The two dimensions with three possible values (i.e., *CL *and *Manner*) have virtually identical rates of agreement, while *KT *has the lowest agreement rate (albeit only by a small amount). This is, however, to be expected - *KT *has 6 possible values and, in many cases, contextual information other than clue expressions is required to determine the correct value. Therefore, it can be a more demanding task than the assignment of other dimensions.

### Annotation discrepancies

We have studied the cases where there is a discrepancy between the two annotators. Whilst a number of these discrepancies are simple annotation errors, in which a particular dimension value was mistakenly selected during the annotation task, other discrepancies occur when a dimension value is identified by means of a clue expression that is not present in the list provided in the guidelines. In some cases, one of the annotators would notice the new clue, and use it to assign an appropriate category, but the other annotator would miss it. In order to minimise the occurrence of such cases, annotators were asked to flag new clue expressions, so that the lists of clue expressions in the guidelines could be updated to be as comprehensive as possible, and so ease the task of accurate annotation.

One of the largest areas of disagreement was between the *KT *categories of *Observation *and *Fact*. For a number of reasons, distinguishing between these types can often be quite tricky, and sometimes there is no clear evidence to suggest which of the categories should be chosen. Events of both types can be indicated using the present tense, and explicit clue expressions are more frequently absent than present. Often, the extended context of the event (possibly including other sentences) has to be considered before a decision can be made. In some cases, it appears that domain knowledge is required to make the correct decision.

In the remainder of this section, we look at some particular cases of annotation discrepancies, some of which appear to be influenced by the expertise of the annotator.

Long sentences seemed to prove more problematic for the biologist annotator, and meta-knowledge information was sometimes missed when there was a large gap between the clue expression and the event trigger. Consider sentence (11), in which the word *indicated *should cause *both *the event with the trigger *prevented *and the event with the trigger *activated *to be annotated with *KT = Analysis*.

(11) *Accordingly, electrophoretic mobility shift assays (EMSAs) **indicated** that pyrrolidine DTC (PDTC) *PREVENTED *NF-kappaB, and NFAT DNA-binding activity in T cells stimulated with either phorbol myristate acetate plus ionophore or antibodies against the CD3-T-cell receptor complex and simultaneously* ACTIVATED* the binding of AP-1*.

Whilst it is straightforward to understand that *indicated *affects the interpretation of the event triggered by *prevented*, it is less easy to spot the fact that it also applies to the event triggered by *activated*, due to the long description of the T cells, which precedes this trigger.

It appears that having some linguistic expertise is an advantage in order to cope with such cases. The biologist would often fail to consider a clue word as potentially affecting the interpretation of an event unless it occurred in close proximity to the event itself. In contrast, the linguist would normally detect long distance dependencies between clue expressions and triggers without difficulty. This is to be expected, given that the linguist is familiar with grammatical rules. However, given the generally high levels of agreement, such complex cases appear to be reasonably rare.

Other annotation discrepancies reveal further differences in the approaches of the annotators. Whilst some grammatical knowledge appears to be advantageous, using a purely grammatical approach to the recognition of meta-knowledge is not always correct. The semantic viewpoint appears to be the one most naturally taken by the biologist annotator, as is evident in sentences such as (12):

(12) *This study **demonstrates **that GC act as a primary* INDUCER* of sialoadhesin expression on rat macrophages, and that the response can be* ENHANCED *by IFN-beta, T cell-derived cytokines, or LPS*.

In (12), we focus on the events triggered by *inducer *and *enhanced*, which are both of type *Positive_Regulation*. The word *demonstrates *is a clue expression for *KT = Analysis*. Taking a purely grammatical approach, the word *demonstrates *affects the interpretation of the verbs *act *and *enhanced*. Of these, only *enhanced *is an event trigger. Accordingly, both annotators marked the event triggered by *enhanced *as *KT = Analysis*. However, the biologist also annotated the *inducer *event with *KT = Analysis*, also marking *demonstrates *as the clue expression. Considering semantics, this is correct - the actual meaning of the first part of the sentence is that *This study demonstrates that GC induces sialoadhesin expression on rat macrophages*.

Example (13) illustrates the need to carefully consider the meaning of words and phrases in the context of the event, as well as simply looking for relevant keywords.

(13) *Changes of any cysteine residue of the hRAR alpha-LBD had **no significant ***INFLUENCE *on the binding of all-trans RA or 9-cis RA*.

In (13), one of the annotators had annotated the *Regulation *event with the trigger *influence *with *Polarity = Negative *(clue word: *no) *and *Manner = High *(clue word: *significant*). However, this is incorrect - it is the word *significant *that is negated, rather than the event itself. As *significant *would normally be a marker of *High *manner, negating it means that it should be treated as a *Low *manner marker. Accordingly, the other annotator correctly identified *no significant *as the clue phrase for *Manner = Low*, with the polarity of the event correctly remaining positive.

The interplay between events in the GENIA event corpus can be complex, especially as events can sometimes occur that have no trigger phrase. The links between different events in a sentence often have to be understood before it can be determined to which of these events a particular piece of meta-knowledge should apply. In such cases, a detailed understanding of the domain could be considered to be an advantage. The following sentence fragment (14) illustrates such a case, in which *absence *constitutes a clue expression for *Polarity = Negative *for one of the events.

(14) *In the **absence **of TCR-*MEDIATED* activation, Vpr* INDUCES* apoptosis*...

Three events were identified as part of the original GENIA event annotation:

1) A *Positive_Regulation *event with the trigger *mediated *(i.e., positive regulation of activation by TCR). At first glance, it is this to event that the negative polarity appears to apply.

2) A second *Positive_Regulation *event, with the trigger *induces *(i.e. positive regulation of apoptosis by Vpr).

3) A *Correlation *event with no trigger, providing a link between events 1) and 2). In fact, it is this third event to which the negative polarity applies. The event conveys the fact that Vpr induces apoptosis even when there is no TRC-mediated activation, indicating that there is *no *correlation between events 1) and 2).

The above examples demonstrate that accurate meta-knowledge annotation can be a complex task, which, according to the event in question, may have to take into account the structure and semantics of the sentence in which the event is contained, as well as the semantics of the event itself, and possibly the interplay between events.

Our inter-annotator agreement results suggest, however, that the annotation task can be accurately undertaken, given appropriate guidelines and training. Furthermore, the results provide evidence that high quality meta-knowledge annotations can be produced regardless of the expertise of the annotator. Although we have highlighted certain cases where either domain knowledge or linguistic expertise appears to be a distinct advantage, neither seems to be a prerequisite. This is in agreement with [13], in which biologist annotators were trained to carry out linguistically-motivated annotation of biomedical events, with good levels of agreement.

## Conclusion

We have designed an annotation scheme to enrich corpora of biomedical events with information about their characterisation or interpretation (meta-knowledge), based on their textual context. The scheme is unique within the field in that it allows detailed meta-knowledge to be annotated at the level of the event, through the use of multiple annotation dimensions. These different dimensions, and the interplay between them, aim to facilitate the training of advanced event extraction systems that can detect various differences between events, both subtle and substantial, which existing systems would fail to recognise.

The scheme is designed to be portable, in order to allow integration with the various different schemes for event annotation that are currently in existence. As a first major effort, our scheme has been applied by 2 external annotators to the largest currently available corpus of biomedical events, i.e., the GENIA event corpus, which consists of 1000 MEDLINE abstracts, annotated with a total of 36,858 annotated events. The annotators achieved inter-annotator agreement rates of between 0.84-0.93 Kappa (according to annotation dimension), demonstrating that high levels of annotation quality and consistency can be achieved by following the annotation guidelines. Furthermore, it appears that, subject to the provision of these guidelines and a suitable training programme, meta-knowledge annotation can be performed to a high standard by annotators without specific areas of expertise, as long as they have a good command of the English language.

An examination of the characteristics of the annotated corpus has revealed that, although all categories within all dimensions have been annotated to a certain extent, their distribution is somewhat skewed, with a heavy emphasis on events that describe observations, relatively few speculative events, and a very low percentage of events that can be attributed to work outside the current study. These results correlate with the general characteristics of scientific abstracts. Although we have so far only applied our scheme to abstracts, it is intended also to be suitable for application to full papers, and we hypothesise that some of the categories of our scheme may be more frequently annotated in this context. For example, the background section of a full paper consists mainly of descriptions of work carried out in previous studies, meaning that a greater proportion of events with *Source = Other *should be observable. The GENIA event annotation scheme is currently being applied to full papers, and it is our intention to apply our meta-knowledge scheme to these papers, both to ensure that the our meta-knowledge scheme is scalable to longer texts, and also to test our hypotheses regarding the different distributions of the annotation dimensions in this context.

As further directions of future work, and inspired by the favourable results of [44] in training a system to recognise several annotation dimensions, we plan to work on the development of a machine learning system that can predict meta-knowledge information for events, trained on our annotated corpus. It is hoped that the comprehensive annotation of clue expressions for the different annotation dimensions, together with the observations we have made about other relevant features, e.g., tense or prototypical positions of particular event types, will constitute useful features that can be used by the system. In addition, we plan to apply our meta-knowledge scheme to event corpora that use different event annotation schemes, such as GREC [13] or BioInfer [15], as well to protein-protein interaction corpora, such as AIMed [52]. Finally, we plan to investigate to what extent our scheme is portable to other scientific domains.

## Authors' contributions

All authors contributed to the production of the manuscript. SA and JM supervised all steps of the work. PT and RN conceived of and designed the annotation scheme, produced the annotation guidelines, performed the annotation of the test corpus, trained the independent annotators and carried out the analysis of the corpus. PT supervised the annotation carried out by the independent annotators. All authors read and approved the final manuscript.
